# Computational Investigation of Advanced Refractive Index Sensor Using 3-Dimensional Metamaterial Based Nanoantenna Array

**DOI:** 10.3390/s23031290

**Published:** 2023-01-23

**Authors:** Sneha Verma, B.M.A. Rahman

**Affiliations:** School of Science and Technology, City University of London, London EC1V 0HB, UK

**Keywords:** nano-antenna, surface plasmon resonance, plasmonic sensitivity, refractive index sensing, Localized surface plasmon resonance

## Abstract

Photonic researchers are increasingly exploiting nanotechnology due to the development of numerous prevalent nanosized manufacturing technologies, which has enabled novel shape-optimized nanostructures to be manufactured and investigated. Hybrid nanostructures that integrate dielectric resonators with plasmonic nanostructures are also offering new opportunities. In this work, we have explored a hybrid coupled nano-structured antenna with stacked multilayer lithium tantalate (LiTaO_3_) and Aluminum oxide (Al_2_O_3_), operating at wavelength ranging from 400 nm to 2000 nm. Here, the sensitivity response has been explored of these nano-structured hybrid arrays. It shows a strong electromagnetic confinement in the separation gap (*g*) of the dimers due to strong surface plasmon resonance (SPR). The influences of the structural dimensions have been investigated to optimize the sensitivity. The designed hybrid coupled nanostructure with the combination of 10 layers of gold (Au) and Lithium tantalate (LiTaO_3_) or Aluminum oxide (Al_2_O_3_) (five layers each) having height, *h*_1_ = *h*_2_ = 10 nm exhibits 730 and 660 nm/RIU sensitivity, respectively. The sensitivity of the proposed hybrid nanostructure has been compared with a single metallic (only gold) elliptical paired nanostructure. Depending on these findings, we demonstrated that a roughly two-fold increase in the sensitivity (*S*) can be obtained by utilizing a hybrid coupled nanostructure compared to an identical nanostructure, which competes with traditional sensors of the same height, (*h*). Our innovative novel plasmonic hybrid nanostructures provide a framework for developing plasmonic nanostructures for use in various sensing applications.

## 1. Introduction

Researchers have become more interested in surface plasmon polaritons (SPPs), since they can provide fresh, remarkable opportunities for the future era of nanotechnologies. Due to the advancement of semiconductor fabrication techniques and electron beam lithography, researchers have lately delved into the manufacture of nano-antennas [1], which range in size from a few hundreds of nanometers to over several microns. To construct effective nano photonic devices [1] with ultra-fast operational speed and the capability to concentrate the electromagnetic field into a region which is significantly narrower than the operating wavelength [2], using SPPs ensures that the objectives of the nanophotonic branch [3] are addressed. SPPs have been used extensively in several technologies, including waveguides [4], modulators [5], nano-lasers [6], nano rings [7], nano wires [8] and nano-antennas [9]. These can be advantageous because of their rapid transient response, compactness, and adjustability in efficiency parameters. However, since metal is dispersive in the visible spectrum, so it must be simulated with an accurate dielectric permittivity function [10,11,12,13,14,15]. Yousafi et al. [16] have suggested a rectangular patch nanostructure to radiate the localized electromagnetic wave power of the hybrid plasmonic waveguide [17,18,19] in which the electromagnetic waves were contained in thin material having very low refractive index in between plasmonic metallic layers. Another purpose of using a hybrid nano antenna system is to achieve the high propagation length. In the traditional single nano antenna system, low efficiency has been seen due to their significant ohmic losses [20,21,22]. To avoid this drawback, a hybrid nano antenna is proposed, which can enhance the performance of the antenna system by reducing the ohmic losses [23,24,25,26]. The separation distance also [27,28,29] plays an important role in enhancing the applied electric field for plasmonic nanostructures [30,31,32,33,34,35,36,37,38,39,40,41,42]. By using the coupled hybrid structure, the electric field confinement can be enhanced and reduce the ohmic losses by using dielectrics (having large refractive index) [43,44,45]. Several studies have been presented to support these arguments using meta-surfaces [46,47], meta-materials [48], architectural color combinations [49], and optical nano antennae [50,51,52,53]. Dielectric and metallic nanostructure combinations have been demonstrated to be able to alter the linear and non-linear far field behaviors of paired structures [54,55,56,57,58,59], in addition to the fact that they emit radio frequencies to work as antennas [60,61]. Recently, in terms of sensitivity, there have been several reports of biosensors using artificially created metamaterials. In 2009, Kabashin et al. reported [62] a sensitivity of 30,000 nm/RIU for two-dimensional porous gold nanorod arrays on a plasmonic hyperbolic metamaterial for biosensor applications. However, despite being miniaturized for commercial biosensing applications, this bulk Kretschmann arrangement is not suitable for more compact integrated photonic sensors. Subsequently, in 2016, Sreekanth et al. [63] reported a similar sensitivity of 30,000 nm/RIU for a grating coupled hyperbolic metamaterial for bulk refractive sensing. However, in the presented work we report on a planar optic design, suggesting good sensitivity which can be more compact, and potentially of lower cost when mass produced.

In this paper, we have proposed a novel hybrid lithium tantalate (LiTaO_3_) or aluminum oxide (Al_2_O_3_) multilayer stacked elliptical paired nanoantenna. These materials are more widely available, less toxic, and thus more appropriate for biological/medical applications. In the case of LiTaO_3_, the magnitude of the spontaneous polarization changes the temperature and disappears at a critical temperature called the Curie temperature (TC). For LiTaO_3_, ferroelectrics are of particular interest for biological applications as their TC is very high (680 °C), which is very far from typical operating temperatures in biological applications. Similarly, Al_2_O_3_ ceramics are being widely used for medical devices, and their biocompatibility is well known and has been reported in recently published articles. Hence, both of these materials used in the proposed device are bio-compatible and can be used in a variety of biomedical and other sensing applications. Recently published studies of the multilayered hybrid plasmonic antenna, which surpasses prior plasmonic waveguides in terms of confinement and propagation losses [24,25], served as the inspiration for the proposed hybrid nanostructures. Unlike the usually presented local field antenna, our proposed nano structure enables an efficient performance even while retaining a very high intensity in the local field. This paper is divided into four sections, where Section II describes the computational design and optimization methods. Section III evaluates the parametric studies of the multi-layer structure. Section IV discusses the effect of the separation distance on the LiTaO_3_ and Al_2_O_3_ stacked nanostructures. Finally, a conclusion and future possibilities are drawn.

## 2. Approaches for Computational Design and Optimization

**Figure 1:**
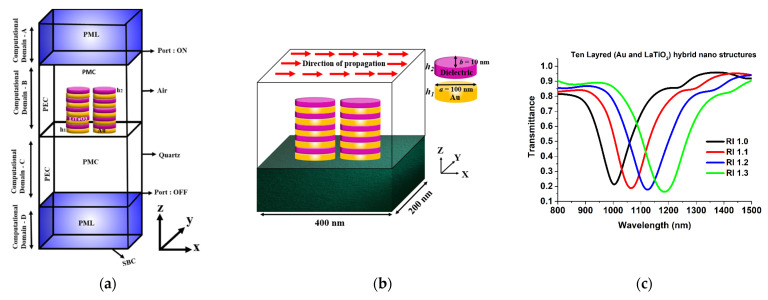
(**a**) Schematic of the computational domain designed on the FEM method enabled commercial software (**b**) Graphical representation of the designed hybrid refractive index sensor (**c**) Transmission spectra of the optimized paired elliptical nano structure with major axis, *a* = 100 nm and minor axis, *b* = 10 nm.

In this paper, the COMSOL Multiphysics software enabled with the finite element method (FEM) in the frequency domain has been used to calculate the plasmonic response and to design the coupled hybrid nano structured antenna, as shown in Figure 1. Figure 1a shows the computational domain, and to reduce the computational time (for designing the whole array of the metamaterial antenna array), we have designed the unit cell and enforced the periodicity in the x and y directions. In the computational domain, the Perfect Magnetic Conductor (PMC) has been used along the *x*-axis and the Perfect Electric Conductor (PEC) has been employed along the *y*-axis. By using these boundary conditions, the whole computation walls will act as a mirror and compute the results for the metamaterial antenna array.

To reduce the back reflection, the Perfect Matched Layer (PML) has been used along the z-direction. The quartz substrate has been optimized 400 × 200 nm^2^ length and width, respectively. A hybrid nano antenna array has been excited by x-polarized light propagating in the z-direction from the top of the antenna array, i.e., the polarization is parallel to the *x*-axis of the antenna dimer. The final design of the 10 layered hybrid sensor system placed on the 400 × 200 nm^2^ quartz is shown in Figure 1a. Figure 1b shows a 3D view of the schematic of the designed computational domain of a hybrid nanostructured antenna array. The dielectric properties of gold have been calculated using the Drude-Lorentz model, as it is based on the movement of the unbounded electrons in the metal that causes the surface plasmon resonance. The material properties of Au have been adopted from Johnson et al. [64]. LiTaO_3_ and Al_2_O_3_ are adopted from Moutzouris et al. and Boidin et al. [65], respectively. We have fixed these values in order to make a quick computation, as the height of the source and the substrate did not affect the sensitivity. After we obtained consistent solutions, we have varied the antenna array parameters, and these are only reported here. In this article we have calculated the sensitivity of the paired hybrid nanoantenna array and compared them with a single metallic nano antenna array in the extremely fine mesh size in order to get stable results.

We then explored the sensitivity to variations in the refractive index of the medium of the 10 total layered (with 5 layers of LiTaO_3_ (or Al_2_O_3_) and gold each) stacked elliptical-shaped paired nano structure, with its minor axis, *b* = 10 nm and major axis, *a* = 100 nm. Linearly *x*-polarized electromagnetic waves in the *z*-direction were used to illuminate these paired nano structures. Through the analysis of transmittance at various refractive index values, its sensitivity has been optimized. Figure 1c displays the transmission spectra for a design specification using various surrounding media (n). Here, the major axis *a =* 100 nm, minor axis, *b* =10 nm, separation distance, *g =* 10 nm and *h*_1_ = 10 nm and *h*_2_ = 10 nm are selected, as are the LiTaO_3_ (or Al_2_O_3_) and gold (Au) thickness, respectively of the stacked nano structure. Since Figure 1b demonstrates a more effective change in the resonating wavelength, it can be employed as a refractive index sensor and is a good contender for biosensing applications. The spectral absorption of the narrow band paired structures can also be modified to match the distinctive absorption spectra of a certain targeted RI in order to identify the targeted medium inside the infrared range. To calculate the sensitivity, the following equation has been used.

(1) $$S=\frac{-\delta \lambda_{res\;}\;}{\delta n_{s}}$$

where, *λ_res_* is the shift in the resonance wavelength and *n_s_* is the surrounding refractive index.

## 3. The Parameterized Investigation of the Multi-Layered Structure

**Figure 2:**
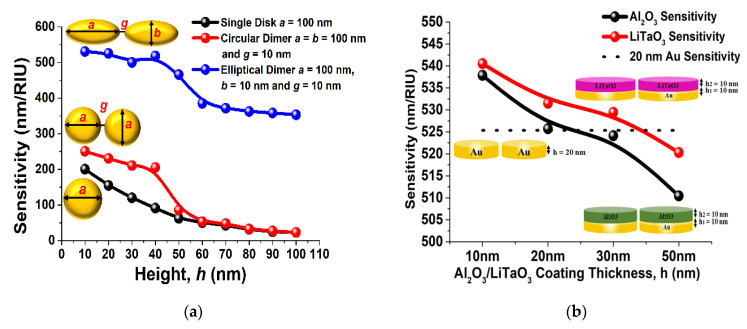
(**a**) The performance of the single, coupled circular and coupled elliptical shaped gold nano antenna array [66] (**b**) The sensitivity performance of the two-layer hybrid nanoantenna array.

In this section, we have analyzed the performance of a hybrid nano structure and compared it with a single metal nano structure. Figure 2a shows the comparative analysis of sensitivity of the single, paired circular and paired elliptical metallic nano structures. Here, a black curve shows that when *h* = 100 nm the sensitivity value was nearly 5 nm/RIU and increases as *h* is reduced, and it reaches nearly 200 nm/RIU when *h* = 10 nm for a single nano disk. The response of the paired circular nano antenna array when *a* = *b* = 100 nm and *g* = 10 nm is shown by a red curve, and the highest sensitivity of 250 nm/RIU was achieved when *h* = 10 nm, which sharply increases for lower *h* values. The sensitivity response of the paired elliptical shaped antenna array is shown by a blue curve when *a* = 100 nm, *b* = 10 nm, and *g* = 10 nm. The blue curve shows the highest sensitivity value of nearly 525 nm/RIU at *h* = 10 nm and it gradually decreases for higher *h* values. In all cases, it can be observed that the highest sensitivity of a single metal dimer can be achieved at nearly 525 nm/RIU when its height is reduced to 10 nm. The motivation of selecting the aspect ratio b/a = 1/10 is based on the study reported in a previous manuscript [66].

Verma et al. [66] have shown that as the separation distance, *g*, decreases, the value of the sensitivity increases, and at *g* = 10 nm, the highest sensitivity value has been achieved. Additionally, the performance of the symmetry has also been discussed with respect to the separation distance, *g*. In this work, we will show that the performance can be further improved by placing a layer of LiTaO_3_ or Al_2_O_3_ on top of the metallic paired nano antenna array. Figure 2b shows the sensitivity comparison of the paired gold elliptical shaped antenna array, where the LiTaO_3_ or Al_2_O_3_ has been stacked on the earlier optimized [66] elliptical dimer with *a* = 100 nm, *b* = 10 nm, *g* = 10 nm, and metal thickness *h*_1_ = 10 nm. A black dashed curve shows a nearly 523.543 nm/RIU sensitivity of single layer gold elliptical dimer antenna array when *h* was kept constant at 20 nm. On the other hand, when Al_2_O_3_ was placed on the top of the paired elliptical shaped antenna array, the sensitivity increases and reaches up to 532 nm/RIU (shown by the black curve). The values increase even more and reaches up to 543 nm/ RIU (shown by a red curve) for LiTaO_3_ for *h*_2_ = 10 nm. From this it can also be stated that as the height *h*_2_, of the LiTaO_3_ and Al_2_O_3_ layer decreases, the sensitivity is increasing. Although it is true that sensitivity increases as the metal or dielectric layer thickness is reduced, getting a very thin layer may bring fabrication uncertainty, and for a fair comparison, the minimum height, *h*_2_ of the LiTaO_3_ and Al_2_O_3_ layer is fixed at 10 nm for further observations.

### 3.1. Performance of the Ten Layered Elliptical Shaped Antenna Array Stacked with Al_2_O_3_ and LiTaO_3_

The sensitivity of the stacked antenna array is next evaluated in this section, where we have shown the sensitivity performance of a multiple layered paired elliptical shaped antenna array designed when *a* = 100 nm, *b* = 10 nm, *g* = 10 nm, and *h* = 100 nm.

**Figure 3:**
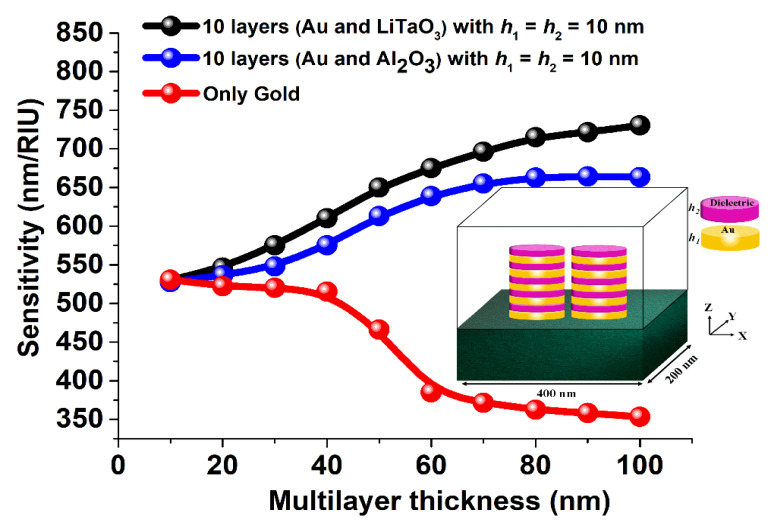
The sensitivity performance of a single metallic (Au) and Al_2_O_3_ and LiTaO_3_ stacked antenna array when total height, *h* = 100 nm with or without stacking.

The red curve in Figure 3 shows the sensitivity values when height, *h*, varied from 10 nm to 100 nm for a single metal elliptical dimer. From this figure it can be observed that at a large value of height, *h* = 100 nm, the sensitivity of the single metal antenna arrays its lowest when a value of nearly 360 nm/RIU was achieved. However, as the height, *h* is reduced to 10 nm, the sensitivity increases and reaches its highest value of nearly 525 nm/RIU. On the other hand, the blue curve shows that as the number of the layers in the stacked antenna array (with Al_2_O_3_) with *a* = 100 nm, *b* = 10 nm, *g* = 10 nm, *h*_1_ = 10 nm, and *h*_2_ = 10 nm is increasing, the sensitivity rather increases when the height of the stacked layer is increasing and reaches up to its saturation point of nearly 660 nm/RIU. In other words, it can be concluded that by using an Al_2_O_3_ stacked antenna array the sensitivity can be enhanced by 1.5 times as compared to a single metallic antenna array keeping *h*_1_ fixed at 10 nm. Similarly, the blue curve in Figure 3 demonstrates that by using a 10 layered LiTaO_3_ stacked antenna array with a = 100 nm, *b* = 10 nm, *g* = 10 nm, *h*_1_ = 10 nm, and *h*_2_ = 10, the sensitivity can be further enhanced by more than two-fold (nearly 730 nm/RIU) as compared to the single gold elliptical paired antenna array. It is worth noting a remarkable more than two-fold increase of the sensitivity and the highest electromagnetic field confinement that has been observed by using the stacked antenna array approach. Hence, such LiTaO_3_ and Al_2_O_3_ stacked plasmonic sensors can detect the small change in the surrounding medium with a sensitivity of about 730 nm/RIU and 660 nm/RIU, respectively, and its sensitivity is expected to increase further by decreasing the height of the individual layers and also the corresponding separation distance.

### 3.2. Study of Field Distribution around the Single Metal and Ten-Layered (5 Pairs of Gold and LiTaO_3_ Stacked) Elliptical Shaped Antenna Array

**Figure 4:**
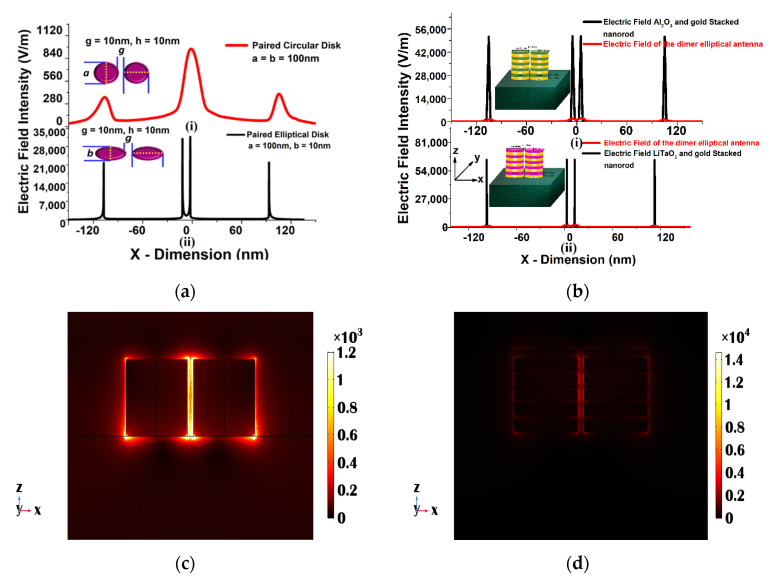
(**a**) Electric field distribution along the *x* plane in the gold elliptical and circular paired structure when *a* = 100 nm, *b* = 10, *g* = 10 nm and *h* = 10 (**b**) Electric field distribution along the *x* plane in the single gold and stacked 10 layers (with LiTaO_3_ or Al_2_O_3_) elliptical paired structure when *a* = 100 nm, *b* = 10, *g* = 10 nm and *h*_1_ = *h*_2_ = 10 nm. (**c**) E_y_, mode field profile at 940 nm wavelength of a single metallic elliptical nano structure when *a* = 100 nm, *b* = 10 nm, and *h* = 100 nm along the *x-z* plane (**d**) Electric field variation at 1000 nm wavelength along the *x-z* plane for a 10 layered LiTaO_3_ stacked nano structure when *a* = 100 nm, *b* = 10 nm, and *h*_1_ = *h*_2_ = 10 nm.

In this section, the performance of the electric field distribution along the single metal and stacked antenna array is discussed. The peak normalized electric field intensity of the single gold circular and elliptical pair was calculated (from COMSOL Multiphysics) as nearly 8.6 × 10^2^ V/m, and 2.9 × 10^4^ V/m reached at the inner edge, as shown in Figure 4a(i) and (ii). It can be noted that the peak fields at the outer edges of the diameter is smaller than the peak field in the gap region. The variation of the electric field, E_x,_ along the *x* direction through the center of the single metallic (gold) elliptical nano structure is shown in Figure 4b by a red curve, which is compared with the Al_2_O_3_ and LiTaO_3_ stacked nanostructured field distribution shown by the black curve (shown in Figure 4b(i) and (ii)). In the case of the Al_2_O_3_ stacked nano structure, the electric field intensity was calculated nearly 5.4 × 10^4^ V/m, which is nearly nine times higher (shown by a black curve in Figure 4b(i)) than that of the single metallic elliptical-shaped nano structure shown by a black curve. However, the electric field intensity even increases further, up to 6.5 × 10^4^ V/m at the inner edges of the LiTaO_3_-stacked elliptical nano structure with *a* = 100 nm, *b* = 10 nm, and *h* = 100 nm, as shown by a black curve in Figure 4b(ii). This value is nearly 10.5 times that of a single gold elliptical nano structure, with *a* = 100 nm, *b* = 10 nm, and *h* = 100 nm. In contrast to a single metallic nano structure, the LiTaO_3_ stacked elliptical nano structure demonstrated in Figure 3 had an improved sensitivity. As a result, it can be regarded as a potential candidate for several bio sensing applications. For single elliptical dimers of height, *h* = 100 nm, Figure 4c shows the mode profile along the center of the *x-z* plane, demonstrating where most of the electric field confinement occurs at the sharp corners and in the separation gap between the two elliptical nano structures. As we have considered the elliptical dimer, the higher electric field exists close to the narrower corners, and the variation of E_y_ along the *x-z* plane for a single elliptical dimer with *h* = 100 nm is shown in Figure 4c. This demonstrates that, due to the absence of a circular symmetry, the electric field intensity was more localized near the sharper corners and at four single metal/ dielectric interfaces at the upper, lower and two sides.

**Figure 5:**
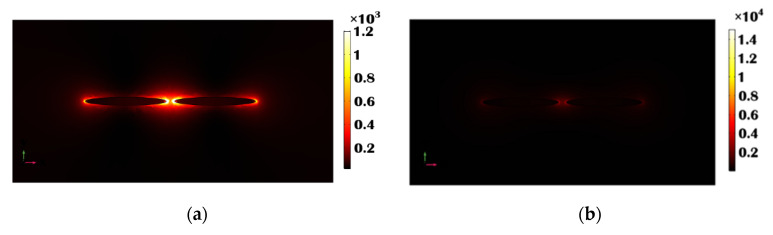
(**a**) E_x_, mode field profile at 940 nm wavelength in the *x-y* plane of a single metallic elliptical nano structure when *a* = 100 nm, *b* = 10 nm, and *h* = 100 nm, where a slice of the horizontal plane is placed at z = 100 nm. (**b**) The electric field profile at the 1000 nm wavelength in the *x-y* plane for a 10 layered LiTaO_3_ stacked nano structure when *a* = 100 nm, *b* = 10 nm, and *h*_1_ = *h*_2_ = 10 nm, where the slice of the horizontal plane is adjusted at *z* = 100 nm.

The electric field distribution along the center of the x-*y* plane of the stacked nanostructure is also shown in Figure 4d, where most of the electric field occurs. From there it can be clearly observed that the electric field intensity is higher and localized at all metal/dielectric interfaces, including the 8 inner metal/dielectric interfaces in the stacked nano structure as compared to the single metallic nano structure. The strong electric field enhancement of the electric field provided by the array of antenna array dimers can be used for a surface-enhanced Raman spectroscopy [67,68,69,70]. Figure 5a displays the E_x_, mode field pattern along the *x-y* plane for an elliptical dimer with a height *h* = 100 nm. It can be observed that the sharp corners and separation gap of the elliptical nanostructure are where most of the electric field confinement occurs. The E_x_-field profile has been shown along the *x-y* plane when *z* = 100 for the LiTaO_3_ stacked antenna, as shown in Figure 5b.

This indicates that the field was more concentrated at the corners and at four single metal/dielectric contacts at *z* = 100 nm, because of the absence of circular symmetry, as shown in Figure 5b. Also, the electric field at *z* = 0 and 50 nm was calculated as 1.0 × 10^2^ V/m and 1.8 × 10^3^ V/m, respectively. Hence, from here it can be stated that the stacked antenna array is a more efficient candidate for the sensing application compared to a single metal antenna array, even with the same other structural dimensions.

## 4. Effect of the Separation Distance on the LiTaO_3_ and Al_2_O_3_ Stacked Nano Structure

**Figure 6:**
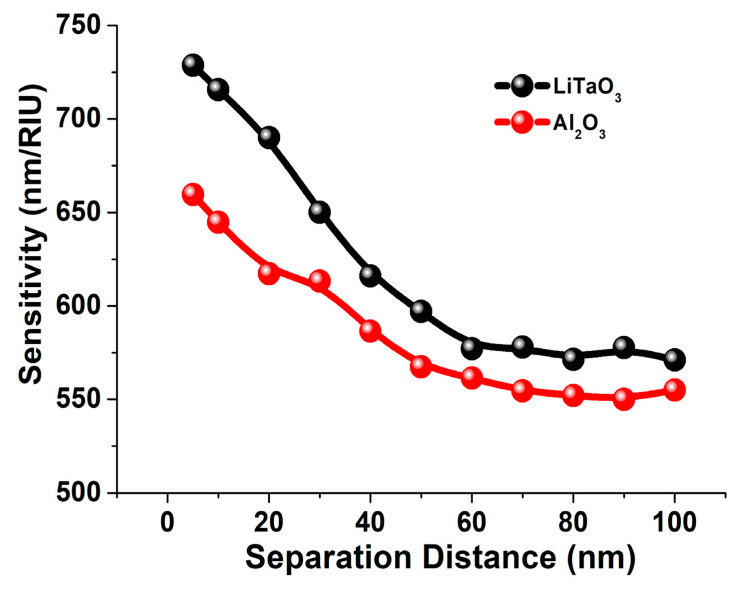
Sensitivity comparison of 10 layered stacked antenna array (5 gold layers with *h*_1_ = 10 nm gold and 5 LiTaO_3_/Al_2_O_3_ layers with *a* =100 nm, b = 10 nm and *h*_2_ (LiTaO_3_ and Al_2_O_3_) = 10 nm) with respect to separation distance.

It is well known that the structural dimensions of the nano structures can enhance the field intensity in the separation gaps, and due to this field enhancement the sensitivity can be affected, so next the performance of the 10 layered LaTiO_3_ and Al_2_O_3_ stacked nano structures was studied. Hence, a 10 layer paired elliptical dimer on the quartz crystal was studied and the sensitivity was calculated when the surrounded medium was covered by the different refractive indices from 1.0 to 1.5. Here, it can be noted that, as shown in Figure 1c for a single case, as the refractive index was increasing, the resonating wavelength was shifting towards the higher range. Figure 6 shows the sensitivity of the LiTaO_3_ and Al_2_O_3_ stacked nano structure when the separation distance, *g* varies from 10 nm to 100 nm. The sensitivity of the 10 layered LiTaO_3_ stacked paired nanostructure is calculated from the slopes of the shift in the transmission spectra from where we observed the linear relationship between the RI values and the plasmonic wavelengths. The R-square error value was calculated as 0.9991 and 0.9817 for the 10 layered LiTaO_3_ and Al_2_O_3_ stacked paired nanostructure, respectively suggesting an almost linear response. Figure 6. clearly shows that at the separation distance, (*g*) = 100 nm, the sensitivity reaches 545 nm/RIU, which is effectively the sensitivity of a single isolated layered elliptical dimer. However, when the separation distance, (*g*) reduced further and reached up to 60 nm, the sensitivity remained nearly constant at 550 nm/RIU. Finally, as the separation distance, (*g*)reduced further to 10 nm, the sensitivity increases rapidly and reaches up to 660 nm/RIU, as shown by the red curve. Similarly, the sensitivity dependence of the LiTaO_3_ stacked antenna array with the separation distance is shown by the black curve.

The highest sensitivity of the LiTaO_3_ stacked antenna array was achieved at nearly 770 nm/RIU when *g* = 10 nm and reduces gradually with the increase in the separation distance. Finally, after *g* = 60 nm the sensitivity remained nearly constant, as shown by the black curve, and at *g* = 100 nm the sensitivity was obtained up to 555 nm/RIU. When the separation distance, *g*, is higher, these metallic antenna arrays are effectively uncoupled and achieved 555 nm/RIU and 545 nm/RIU sensitivity when they could be considered as two isolated antenna arrays. However, as the separation distance, *g* is reduced, these two isolated antenna arrays are now coupled, and they formed an effective dimer and their sensitivity reached up to 660 nm/RIU and 770 nm/RIU for the hybrid Al_2_O_3_ and LiTaO_3_ structure, respectively. Thus, it is demonstrated here that the sensitivity of the hybrid LiTaO_3_ and Al_2_O_3_ paired nano structure is always higher than that of a single metallic nano structure. Tsai et al. [71], reported that by using a coupled nano ring, the sensitivity can be enhanced by up to 50%, but our work shows that for an elliptical nano structure using a stacked antenna array nanostructure. The sensitivity values can be further increased by more than 150% while using a much smaller overall size of the antenna array compared to [66]. The change in angle of incidence can produce the TP resonance mode through the reflectance spectrum, and thus we have compared our work with the Tamm sensors shown in Table 1 below.

Hence, this can be an attractive method for detecting the heavy metals, biochemicals, air quality, and water purity, and this is more efficient and cost-effective (if they are fabricated in bulk) and opens up new pathways for both healthcare and environmental monitoring applications. The comparision between the proposed work and the other metallic and hybrid structures are shown in Table 2.

The proposed hybrid nano antenna system has shown very high sensitivity, and this can be enhanced furthermore by using small geometrical dimensions, but we have suggested suitable dimensions which can be experimentally fabricated. There are several lithographic methods that are available to fabricate such small nano structures as those which are mentioned in the literature [72,73,74,75,76,77,78,79,80,81]. We cannot avoid the fact that to design the smaller structure can be challenging; however, the masking method can work well in this scenario. To avoid further experimental complexity, we have used the same dimensions of the gold and dielectric layer. The asset of the proposed sensor is that it can be used as a stand-alone device with the great amount of stability, as we are proposing that the sensing on the substrate and the material used in the sensing device are highly biocompatible and stable so it can be good to consider these kind of sensing devices.

## 5. Conclusions

In conclusion, we have reported a study of a hybrid (LiTaO_3_ and Al_2_O_3_) stacked metallic nano plasmonic sensor. The designed and optimized sensor with *a* = 100 nm, *b* = 10 nm, *g* = 10 nm, *h*_1_ = 10 nm, and *h*_2_ = 10 nm has been evaluated in various surrounding refractive indices from 1.0 to 1.5 to calculate their corresponding sensitivity. The transmission, absorption, reflection spectra and modal field profiles have also been calculated to observe the sensor performance. The designed hybrid sensor has been compared with a single metallic nanoantenna array when *a* = 100 nm, *b* = 10 nm, *g* = 10 nm, and *h* = 100 nm to observe the sensitivity enhancement. From the aforementioned results, it can be stated that the sensitivity can be enhanced by nearly 1.5 times by using an Al_2_O_3_ stacked antenna array and by more than two times by using LiTaO_3_. It has also been shown that the sensitivity can be further increased by reducing the metal height, *h*_1,_ and the dielectric height, *h*_2_, or the separation distance, *g*. But for a fair comparison, the values of these are taken as 10 nm. The normalized electric field intensity of the LiTaO_3_ and Al_2_O_3_ stacked antenna array were stronger, at nearly 6.5 × 10^4^ V/m and 5.4 × 10^4^ V/m, respectively, which was approximately 10.5 times more than the single metallic nanostructure for LiTaO_3_ and nine times more than the Al_2_O_3_ stacked antenna array. The proposed nano-enhanced antenna’s sensitivity is demonstrated by the use of a full-wave electromagnetic simulation. Our suggested nano-antenna array may be used for different nano inter- and intra-chip photonic sensor systems to develop cutting-edge detecting devices for measuring the quality of water, air, and soils. Furthermore, due of its wide frequency coverage, this suggested antenna array may be employed for biosensing, optical energy harvesting (also known as nano-rectenna (where the top performer rectenna has used oxide/metal bilayer plasmonic antenna [82] or Nantenna and THz Metasurface-mediated nano-biosensors [83]) and various artificial intelligence based [84,85] optical sensing applications.

## Figures and Tables

**Table 1:** Comparision between proposed work with the Tamm sensors.

S.NO.	The Designed Structure	Sensitivity	Sensing Materials	Ref.
1.	1D porous silicon photonic crystal fluid sensor based on TP resonance	5018 nm/RIU	Different fluids	[72]
2.	1D photonic crystal as a gas sensor based on TP resonance	273 nm/RIU	Toluene	[73]
3.	Experimental gas sensor based on TP resonance	70 nm	Organic vapors	[74]
4.	Metallo-dielectric heterostructure configuration based on TP resonance	970 nm/RIU	Different liquids	[75]
5.	Theoretical gas sensor using photonic crystal cavity based on TP resonance	450 nm/RIU	Methane gas	[76]
6.	Theoretical elliptical shaped plasmonic nano antenna array	770 nm/RIU	Water and gases from RI 1.0 to RI 1.7	This work

**Table 2:** Comparision between the proposed work and the other metallic and hybrid structures.

S.NO.	Metal	Dielectric	Shape	Sensitivity	FWHM	Ref.
1.	Silver	GaP	Ring and Heptamer	550 nm/RIU	82.4	[77]
2.	Aluminum	SiO_2_	Bow-Tie	497 nm/RIU	-----	[78]
3.	Gold	SiO_2_/SiC	Photonic Crystal	5.4 nm	-----	[79]
4.	Silver	Si/SiO_2_	Elliptical and MMI waveguide	550 nm/RIU	1.947	[80]
5.	Silver	Si	Ring	636 nm/RIU	------	[81]
6.	Gold	LiTaO_3_/Al2O_3_	Elliptical Stacked	770 nm/RIU	76.4	This Work

## Data Availability

The supporting information can be found from the corresponding author.
